# Total Syntheses of Phleghenrines A and C

**DOI:** 10.1021/acs.orglett.3c01784

**Published:** 2023-07-11

**Authors:** Xinpei Cai, Lei Li, Ye-Cheng Wang, Jianhan Zhou, Mingji Dai

**Affiliations:** † Department of Chemistry and Center for Cancer Research, Purdue University, West Lafayette, Indiana 47907, United States; § Department of Chemistry, Emory University, Atlanta, Georgia 30322, United States

## Abstract

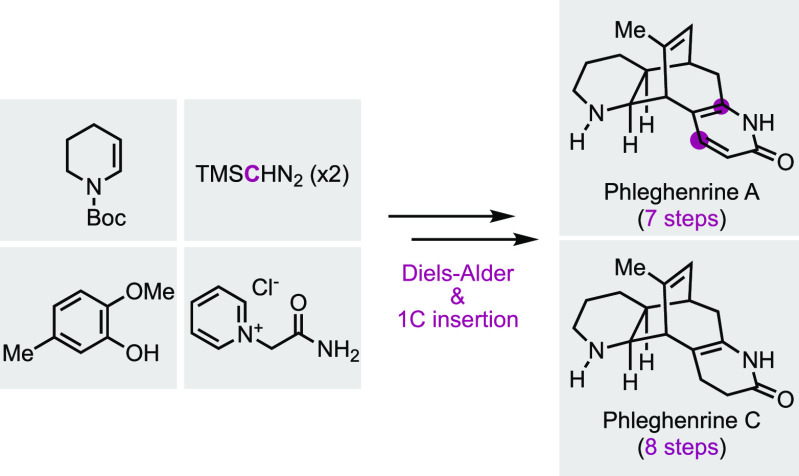

Herein, we report the total syntheses of phleghenrines A and C from commercially available starting materials in 7 and 8 steps, respectively. Notable steps include an inverse electron-demand Diels–Alder reaction between a masked *o*-benzoquinone and a *N*-protected enamine to prepare one key intermediate with a bicyclo[2.2.2]octenone core, a Büchner–Curtius–Schlotterbeck one-carbon insertion to expand the bicyclo[2.2.2]octenone to a bicyclo[3.2.2]nonenone, and Trauner’s modified 2-pyridone synthesis to install the 2-pyridone moiety.

Phleghenrines A–D (**1**–**4**, Figure 1) and neophleghenrine A (**5**) were isolated by Zhao and co-workers in 2016 from *Phlegmariurus henryi* Ching, part of the Huperziaceae.^1^ In addition to the phleghenrine molecules, *Phlegmariurus henryi* Ching was reported to produce huperzine A (**6**) and B (**7**), two famous *lycopodium* alkaloids with potent acetylcholinesterase inhibition activity. Huperzine A has been used as a new drug to treat Alzheimer’s disease in China and as a dietary supplement in the United States.^2^ It was evaluated in human clinical trials to treat traumatic brain injury as well. The phleghenrine molecules also demonstrated inhibitory activity against acetylcholinesterase with phleghenrine A (**1**) and D (**4**) as the most potent family members (IC_50_ = 4.91 μM for **1** and 4.32 μM for **4**). Notably, the phleghenrines showed low or no inhibition activity against butylcholinesterase; thus, they may exhibit less side effects such as hepatoxicity. While phleghenrines A, B, and D share a similar 2-pyridone moiety as huperzines A and B as well as lyconadins A and C,^3^ another two *lycopodium* alkaloids with neurotrophic activity, the phleghenrines have a distinct and complex tetracyclic skeleton featuring a bicyclo[3.2.2]nonene core. Their novel chemical structures and potent acetylcholinesterase inhibition activity render them promising lead compounds for the drug discovery efforts in searching for effective treatment of Alzheimer’s disease and other related neurodegenerative disorders. However, the low isolation yield (<0.0003%) is a major hurdle for their further biomedical development. In 2019, Sarpong and co-workers reported their synthetic studies to construct the [3.2.2] bicycles of the phleghenrines.^4^ While this manuscript was in preparation, She and co-workers reported their total syntheses of phleghenrines A and C in 19 and 18 steps, respectively, from a known diene, which requires 4 steps to prepare.^5^ Herein, we reported our total syntheses of phleghenrines A and C in 7 and 8 steps, respectively.^6^

**Figure 1:**
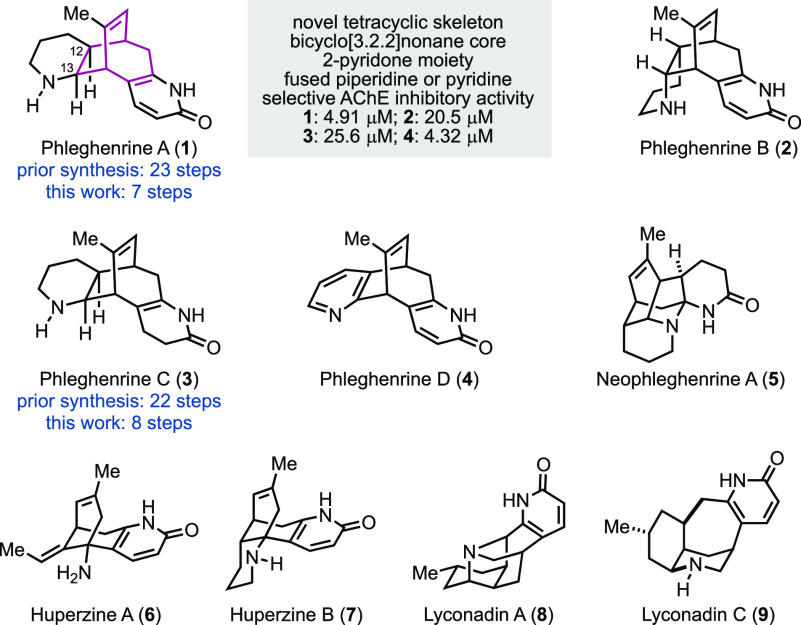
Phleghenrines and related alkaloids.

Our continued interest^7^ in the total synthesis and biological studies of *lycopodium* alkaloids with the therapeutic potential to treat various neurodegenerative disorders prompted us to pursue the total synthesis of the phleghenrine alkaloids. Retrosynthetically (Figure 2), similar to our total synthesis of lyconadins A and C,^7a^ we decided to install the 2-pyridone moiety in the end by using the Fukuyama-Yokoshima 2-pyridone synthesis.^8^ Thus, tricyclic α-methylene ketone **11** with a bicyclo[3.2.2]nonene core was needed and could be synthesized from ketone **13** via a sequence of methylenation and allylic oxidation. In comparison to the bicyclo[3.2.2]nonene ring systems, the bicyclo[2.2.2]octene ring systems are much more accessible via reactions, including the Diels–Alder cycloaddition. With this in mind, we practiced a one-carbon deletion retrosynthetically and proposed intermediate **14** with a bicyclo[2.2.2]octene core as the precursor of **11**. In the forward sense, a one-carbon insertion protocol is needed. Accordingly, we envisioned an approach involving a Büchner–Curtius–Schlotterbeck one-carbon homologation with (trimethylsilyl)diazomethane to convert the cyclohexenone to a cycloheptenone (**14** → **13**).^9^ Retrosynthetically, an acetal functional group addition strategy at this stage would lead to compound **15** as a precursor of **14**. We planned to assemble **15** via an inverse electron-demand Diels–Alder reaction between masked *o*-benzoquinone (MOB) **16** and *N*-protected enamine **17**.^10^ MOB **16** could be synthesized via an oxidative dearomatization of commercially available 2-methoxy-5-methylphenol (**18**), and **17** could be prepared via a formal dehydrogenation of piperidine derivative **19**(11) or reduction of the corresponding δ-valerolactam **20**.^12^

**Figure 2:**
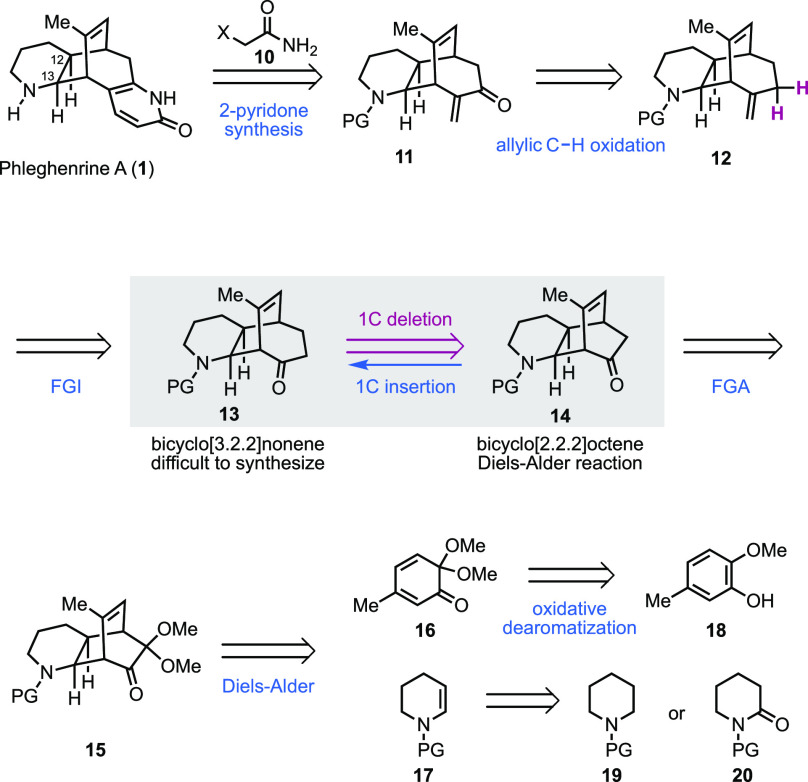
Retrosynthetic analysis.

Our synthesis started from commercially available 2-methoxy-5-methylphenol (**18**) and known Boc-protected enamine **22** (Scheme 1). The latter can be synthesized in one step from commercially available *N*-Boc-protected δ-valerolactam^12^ or *N*-Boc-protected piperidine.^11^ We first tried to generate MOB **16** via an oxidative dearomatization of **18** with phenyliodonium diacetate in MeOH. While **16** could be formed smoothly, it underwent spontaneous Diels–Alder dimerization and cannot be isolated. Instead, dimeric product **21** was obtained. Efforts were then directed to trap MOB **16** in situ with dienophile **22** to form **23** but were unsuccessful. Since the Diels–Alder dimerization of **16** is reversible, we also explored the possibility of generating **16** via a retro-Diels–Alder reaction from **21** and trapping it in situ with **22**. Unfortunately, no desired product **23** was observed, as well. To avoid the problematic MOB dimerization, we decided to install a bromide at the C-4 position of **16** (see **25**). The bromide has been shown to block the dimerization and can be removed later.^13^ Thus, known compound **24** was prepared in 96% yield by bromination of **18** with a reported procedure using *N*-bromosuccinimide (NBS) in AcOH.^14^ As expected, oxidative dearomatization of **24** with phenyliodonium diacetate in MeOH gave known Br-MOB **25**, which can be purified and characterized. After evaporation of MeOH, **22** was added, and the resulting neat mixture was heated at 140 °C to deliver the Diels–Alder adducts via a one-pot procedure in 85% yield as a separable 4.4:1 *endo*/*exo* (**26**/**27**) mixture. The bromide, after serving its purpose of blocking the MOB dimerization, was then removed with SmI_2_, which also removed the extra dimethyl acetal at the α-position of the ketone. Tricyclic compound **28** with a bicyclo[2.2.2]octenone core was obtained in 83% yield.

**Scheme 1:**
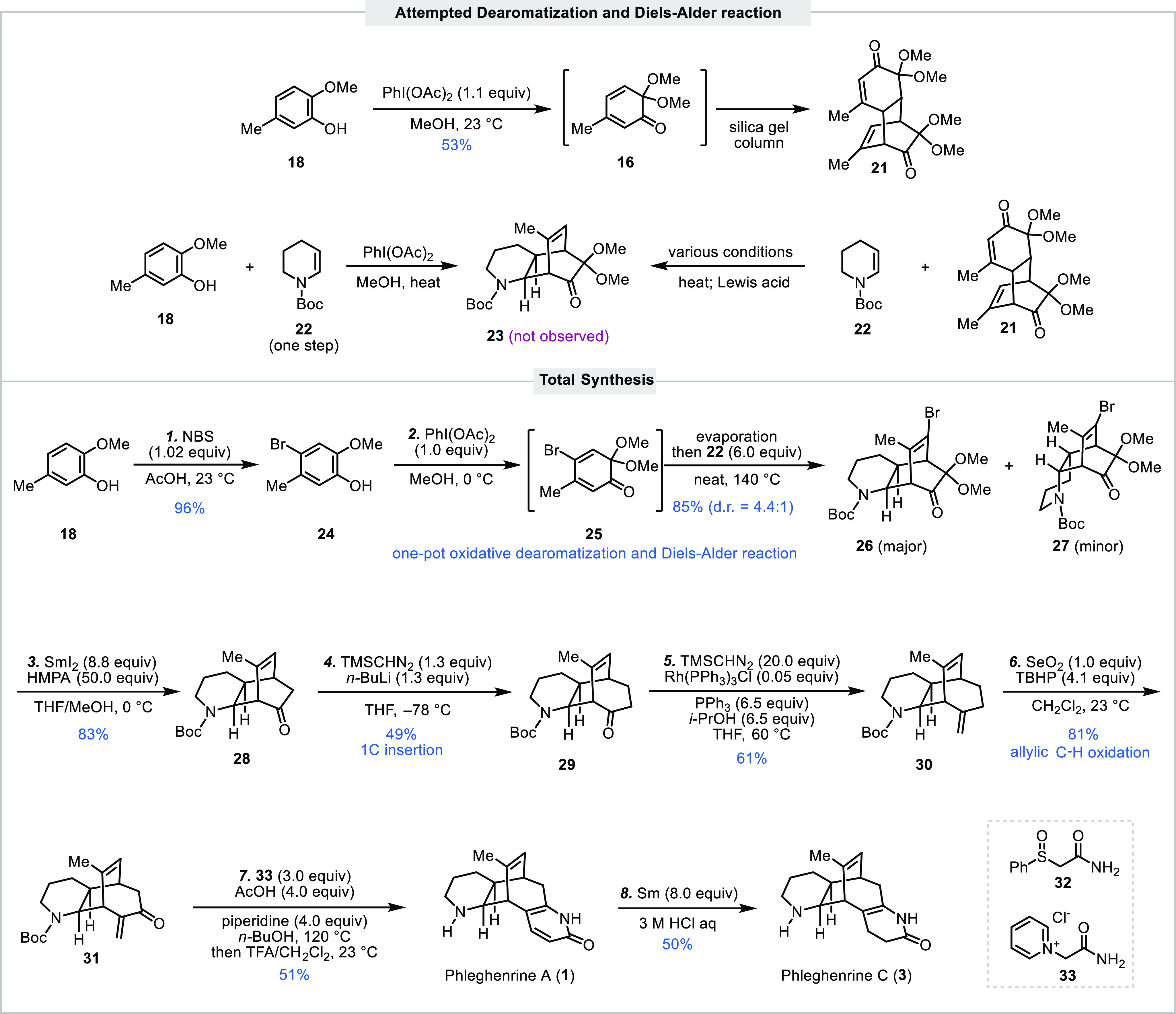
Total Syntheses of Phleghenrines A and C

We then moved on to explore the Büchner–Curtius–Schlotterbeck one-carbon insertion and were happy to learn that desired product **29** with a bicyclo[3.2.2]nonenone core could be obtained in 41% yield by exposing **28** to (diazo(trimethylsilyl)methyl)lithium generated from treating (trimethylsilyl)diazomethane with *n-*BuLi in THF at low temperature. Compound **29** was further advanced to α-methylene ketone **31** in two steps for the next 2-pyridone synthesis. The Wittig one-carbon homologation was realized by using the Lebel modification, which releases methylenetriphenylphosphorane catalytically from the Wilkinson’s catalyst, PPh_3_ and trimethylsilyldiazomethane.^15^ Product **30** was obtained in 61% yield. Notably, the yield from conventional Wittig methylenation is much lower (36%). Allylic C–H oxidation with a combination of SeO_2_ and *tert*-butyl hydroperoxide (TBHP) occurred smoothly and delivered **31** in 81% yield. The last step is to install the 2-pyridone moiety, which turned out to be nontrivial. We first explored the Fukuyama-Yokoshima 2-pyridone synthesis protocol using 2-(phenylsulfinyl)acetamide **32**. After comprehensive explorations, we were able to get the desired product phleghenrine A (**1**) via one-pot Boc removal, but the yield never went above 10%. Acetamide derivative **33** was used by Trauner and co-workers in their lycoposerramine T total synthesis to build the corresponding 2-pyridone.^16^ Thus, we explored Trauner’s protocol and were able to achieve a one-pot 2-pyridone synthesis and Boc-deprotection to synthesize phleghenrine A (**1**) in 51% yield. We further demonstrated that phleghenrine A (**1**) could be partially reduced to phleghenrine C (**3**) with Sm in HCl in modest yield.^17^ Overall, from commercially available starting materials, phleghenrine A (**1**) and phleghenrine C (**3**) were prepared in 7 and 8 steps, respectively, which are significantly shorter than the syntheses (23 and 22 steps) reported recently by She and co-workers.^5^

In summary, concise total syntheses of the structurally novel and scarce *lycopodium* alkaloids phleghenrine A (**1**) and phleghenrine C (**3**) with potent and selective acetylcholinesterase inhibition activities were achieved. The combination of an inverse electron-demand Diels–Alder reaction and a Büchner–Curtius–Schlotterbeck one-carbon insertion enabled an efficient construction of the bicyclo[3.2.2]nonene core of the phleghenrine alkaloids. Other key steps include a Lebel-modified Wittig olefination, allylic C–H oxidation, and Trauner’s modified 2-pyridone synthesis. This current synthetic route could potentially be adapted for phleghenrine analogue synthesis, thus facilitating further biological evaluations of the phleghenrine alkaloids.

## Data Availability

The data underlying this study are available in the published article and its Supporting Information.

## Supporting Information Available

Experimental procedures and spectra data (PDF)
