# Supplementary material for: Total Syntheses of Phleghenrines A and C
Source: Org Lett. 2023 Jul 11;25(28):5258–61. doi: 10.1021/acs.orglett.3c01784 (PMC10367062; doi:10.1021/acs.orglett.3c01784)

Supporting Information

Total Syntheses of Phleghenrines A and C

Xinpei Cai,[a,c] Lei Li,[a,b,c] Ye-Cheng Wang,[a] Jianhan Zhou,[a] and Mingji Dai*[a,b]

[a]Department of Chemistry and Center for Cancer Research, Purdue University, West Lafayette, IN 47907, United States

[b]Department of Chemistry, Emory University, Atlanta, GA 30322, United States

[c]Contributed equally.

Correspondence and requests for materials should be addressed to M. D. (email: mingji.dai@emory.edu)

Table of Contents

Part 1. Experimental procedures and spectra data...S-3

Part 2. References..S-12

Part 3. ^{1}H and ^{13}C NMR spectra...S-13

Part 1. Experimental procedures and spectra data.

General Methods: All reactions sensitive to air or moisture were conducted under argon atmosphere in dry and freshly distilled solvents under anhydrous conditions, unless otherwise noted. Anhydrous tetrahydrofuran (THF), dichloromethane (DCM), Dimethylformamide (DMF) and toluene were purified by passing the pre-degassed solvents through activated alumina columns. All other solvents and reagents were used as obtained from commercial sources (Aldrich, TCI, Alfa Aesar, Acros) without further purification unless otherwise noted. Room temperature is around 23 °C. Flash column chromatography was performed using silica gel (230-400 mesh). Thin layer chromatography (TLC) was performed using glass-backed silica plates (SiliCycle). NMR spectra were recorded on a Bruker AV-500 or Bruker AVANCE III HD 600 spectrometer at room temperature. Chemical shifts (δ) were given in ppm with reference to the solvent signal [^{1}H NMR: $CDCl_3$ (7.26), CD_3OD (3.31); ^{13}C NMR: $CDCl_3$ (77.16), CD_3OD (49.00)]. ^{1}H NMR data were reported as follows: chemical shifts (δ ppm), multiplicity (s = singlet, d = doublet, t = triplet, q = quartet, quin (p) = quintuplet, m = multiplet, br = broad), coupling constant (Hz), and integration. ^{13}C NMR data were reported in terms of chemical shift and multiplicity. High-resolution mass measurements for compound characterization were carried out using a Waters SYNAPT G2-Si system with QuanTof analyzer or an Agilent 6550 QTOF system. IR data were recorded on a Thermo Nicolet iS50 FT-IR.

Experiment procedure

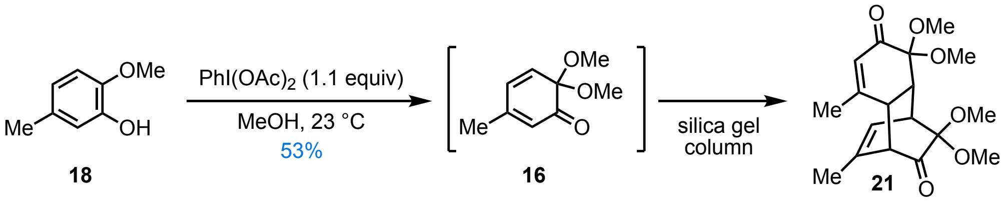

To a solution of commercially available 2-methoxy-5-methylphenol **18** (100.0 mg, 0.724 mmol, 1.0 equiv) in methanol (7.2 mL) was added $PhI(OAc)_2$ (256.4 mg, 0.796 mmol, 1.1 equiv) at room temperature. After the reaction was stirred for 30 min, the mixture was concentrated *in vacuo*. Then the crude was subjected to flash column (Hexane/EtOAc = 6/1) to afford Diels-Alder adduct dimer **21** (136.0 mg, 53%) as white solid, which matched with literature report.[1]

Compound **21**:

^{1}H NMR (500 MHz, CDCl$_3$): δ 5.92 (m, 1H), 5.82 (dt, J = 6.7, 1.8 Hz, 1H), 3.43 (s, 3H), 3.38 (s, 3H), 3.23 (s, 3H), 3.22 – 3.10 (m, 3H), 3.05 (s, 3H), 3.01 (dd, J = 6.7, 1.6 Hz, 1H), 1.96 (d, J = 1.4 Hz, 3H), 1.64 (d, J = 1.6 Hz, 3H);

^{13}C NMR (126 MHz, CDCl$_3$): δ 202.5, 193.2, 156.2, 137.6, 126.6, 124.8, 98.7, 94.9, 57.9, 50.8, 50.2, 49.6, 49.0, 43.4, 39.9, 38.8, 22.3, 21.6;

IR (film): 2942, 2839, 1736, 1696, 1636, 1444, 1385, 1317, 1247, 1216,1140, 1118, 1094, 1054, 1009 cm^{-1};

HRMS (APCI): *m/z* calc. for $C_{18}H_{25}O_6{}^{+}$ [M+H]$^{+}$: 337.1651, found 337.1667.

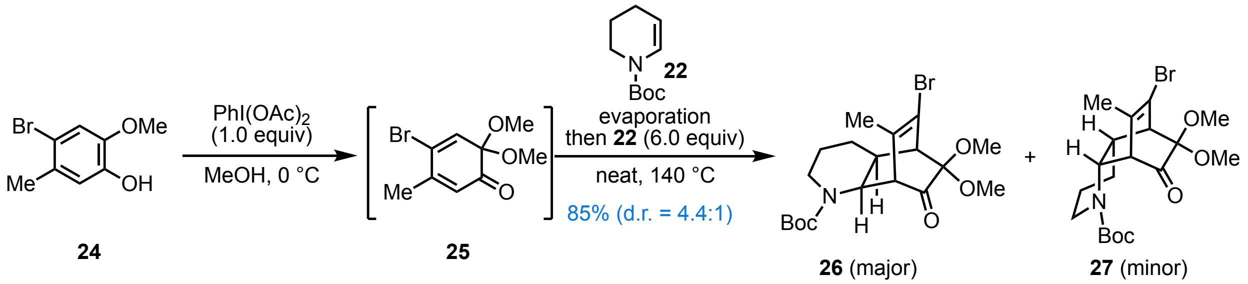

24 **25** **26** (major) **27** (minor)

To a solution of 4-bromo-2-methoxy-5-methylphenol **24**[2] (100.0 mg, 0.461 mmol, 1.0 equiv) in methanol (4.5 mL) was added PhI(OAc)$_2$ (148.4 mg, 0.461 mmol, 1.0 equiv) at 0 °C. The yellow solution was stirred for 30 min at 0 °C. The mixture was concentrated *in vacuo*. To the crude 4-bromo-masked-*o*-benzoquinone was added dienophile **22**[3] (506.5 mg, 2.76 mmol, 6.0 equiv) directly at room temperature and the mixture was heated to 140 °C at neat condition for 3 h. The crude was subjected to flash column (Hexane/EtOAc = 6/1) to remove extra dienophile **22** and then subjected to flash column (CH$_2$Cl$_2$/Et$_2$O = 50/1) to give major Diels-Alder adduct **26** (136.0 mg, 69%) as colorless oil and minor Diels-Alder adduct **27** (30.9 mg, 16%) as colorless oil. (Note: the Diels-Alder reaction was also conducted at 1.5-gram scale at 100 °C, which gave 81% yield of **26** and **27** in 2.1:1 diastereoselectivity.)

Compound **26**:

^{1}H NMR (500 MHz, CDCl$_3$): δ 4.35 (d, J = 9.8 Hz, 1H), 4.01 – 3.90 (m, 1H), 3.35 (s, 3H), 3.30 (s, 3H), 3.25 (s, 1H), 3.18 (d, J = 3.1 Hz, 1H), 2.77 (tdd, J = 10.3, 7.5, 3.0 Hz, 1H), 2.42 (t, J = 12.1 Hz, 1H), 1.93 – 1.88 (m, 1H), 1.86 (s, 3H), 1.67 – 1.61 (m, 1H), 1.51 (m, 1H), 1.45 (s, 10H), 1.35 (m, 1H);

^{13}C NMR (126 MHz, CDCl$_3$): δ 197.2, 154.7, 132.2, 119.2, 93.9, 80.4, 61.8, 54.6, 50.5, 50.3, 48.8, 41.2, 32.5, 28.4, 25.4, 23.2, 20.2;

IR (film): 2973, 2942, 1742, 1688, 1453, 1376, 1365, 1316, 1279, 1252, 1156, 1110, 1089, 1053, 1014 cm^{-1};

HRMS (ESI): *m/z* calc. for $C_{19}H_{28}BrNO_5Na^+$ [M+Na]$^+$: 452.1043/454.1025, found 452.1041/454.1021.

Compound **27**:

^{1}H NMR (600 MHz, $CDCl_3$): δ 4.09 (dd, *J* = 12.1, 2.9 Hz, 1H), 3.89 (d, *J* = 12.7 Hz, 1H), 3.52 (d, *J* = 2.8 Hz, 1H), 3.39 (s, 3H), 3.30 (s, 3H), 3.13 (d, *J* = 2.8 Hz, 1H), 2.64 (ddd, *J* = 13.6, 11.7, 2.7 Hz, 1H), 2.57 (dddd, *J* = 11.6, 10.1, 8.5, 2.8 Hz, 1H), 2.09 (dddd, *J* = 13.8, 11.9, 8.5, 5.3 Hz, 1H), 1.87 (s, 3H), 1.92 – 1.81 (m, 1H), 1.69 – 1.61 (m, 1H), 1.47 (s, 9H), 1.35 – 1.24 (m, 1H);

^{13}C NMR (150 MHz, $CDCl_3$): *δ* 201.6, 155.2, 133.2, 119.4, 96.4, 80.2, 60.1, 53.7, 52.2, 50.4, 49.5, 41.8, 30.1, 28.5, 23.2, 22.6, 19.2;

IR (film): 2936, 1741, 1690, 1456, 1365, 1273, 1160, 1094, 1057, 1011 cm^{-1};

HRMS (APCI): *m/z* calc. for $C_{19}H_{29}BrNO_5^+$ [M+H]$^+$: 430.1224/432.1203, found 430.1245/432.1223.

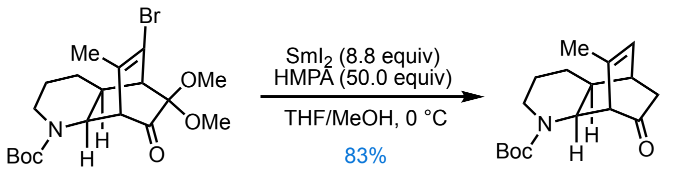

26 **28**

SmI_2 solution in THF was freshly prepared:

To a solution of samarium (11.77 g, 78.3 mmol, 2.0 equiv) in THF (368.4mL) was added iodine (9.95 g, 39.2 mmol, 1.0 equiv) at room temperature. The mixture was heated at 60 °C overnight. The SmI_2 solution was used directly as 0.107 M in THF after cooling to room temperature.

To a solution of ketal **26** (1.79 g, 4.16 mmol, 1.0 equiv) in THF/MeOH (20:1, 41.6 mL) was added HMPA (35.8 mL, 205.8 mmol, 50.0 equiv) at room temperature. The resulting mixture was bubbled with argon (balloon) for 20 min. The freshly prepared SmI_2 was added dropwise at 0 °C until the color of the solution changed to purple (about 343 mL SmI_2 (0.107 M in THF) used, 36.6 mmol, 8.8 equiv). The resulting mixture was stirred for 5 min before it was quenched with air flow and the color changed from purple to yellow. Most THF was concentrated *in vacuo*. Then

EtOAc was added and the solid in system was removed by a short celite column. The filtrate was concentrated *in vacuo*. The resulting mixture was subjected to flash column (Hexane/EtOAc = 4/1) to afford ketone **28** (1.00 g, 83%) as pale yellow oil.

Compound **28**:

^{1}H NMR (500 MHz, CDCl$_3$): δ 6.11 (dt, J = 6.5, 1.8 Hz, 1H), 4.31 (d, J = 10.2 Hz, 1H), 3.94 (d, J = 12.3 Hz, 1H), 3.15 (s, 1H), 2.82 (m, 1H), 2.49 (m, 1H), 2.38 (m, 1H), 2.04 (dd, J = 5.3, 2.9 Hz, 2H), 1.89 (m, 1H), 1.83 (d, J = 1.7 Hz, 3H), 1.61 (m, 1H), 1.48 (s, 9H), 1.45 – 1.35 (m, 2H);

^{13}C NMR (126 MHz, CDCl$_3$): δ 210.1, 155.2, 135.4, 130.0, 80.2, 60.6, 50.5, 41.3, 38.9, 38.1, 35.8, 28.6, 27.4, 23.1, 21.2;

IR (film): 2936, 1727, 1686, 1444, 1376, 1364, 1322, 1300, 1253, 1150, 1114, 1054, 1007 cm^{-1};

HRMS (ESI): *m/z* calc. for $C_{17}H_{25}NO_3Na^+$ $[M+Na]^+$: 314.1726, found 314.1729.

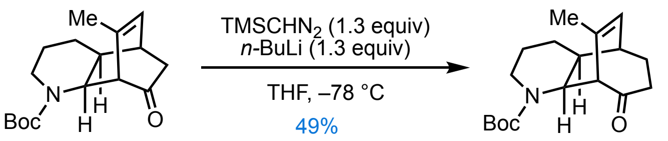

28 **29**

Ketone **28** solution preparation:

Ketone **28** (194 mg, 0.67 mmol, 1.0 equiv) was dissolved in strictly anhydrous THF (1.6 mL) under argon atmosphere.

A vial equipped with (trimethylsilyl)diazomethane (10% in hexanes) (1.46 mL, 0.87 mmol, 1.3 equiv) and a magnetic stirring bar was charged with anhydrous THF (10.0 mL) under argon atmosphere at room temperature. *n*-BuLi (2.5 M in hexane) (0.35 mL, 0.86 mmol, 1.3 equiv) was added at –78 °C. The reaction mixture was stirred for 30 min. Then the solution of ketone **28** was added and the reaction mixture was stirred for 30 min at the same temperature. MeOH (0.15 mL) was added, and the reaction mixture was stirred for 30 min. All process was under argon atmosphere at –78 °C. The reaction was quenched by addition of water. The reaction mixture was extracted with EtOAc. The combined organic layer was washed with water, washed with brine, dried over anhydrous Na_2SO_4 and concentrated *in vacuo*. The residue was purified by flash column (Hexane/EtOAc = 6/1) to afford rearranged ketone product **29** **(**100 mg, 49%) as pale yellow oil.

Compound **29**:

^{1}H NMR (500 MHz, CDCl$_3$): δ 5.91 (d, J = 7.5 Hz, 1H), 4.68 (s, 1H), 4.00 (s, 1H), 2.66 (s, 1H), 2.57 (m, 2H), 2.46 – 2.35 (m, 2H), 2.32 – 2.23 (m, 1H), 2.00 (m, 1H), 1.91 (m, 1H), 1.79 (s, 3H), 1.70 – 1.64 (m, 1H), 1.62 (s, 1H), 1.49 – 1.47 (m, 1H), 1.45 (s, 9H), 1.41 (m, 1H);

^{13}C NMR (126 MHz, CDCl$_3$): δ 204.6, 155.2, 134.1, 128.5, 80.3, 63.4, 50.2, 40.4, 38.0, 37.8, 37.2, 29.9, 28.5, 27.6, 23.8, 21.5;

IR (film): 2931, 1689, 1449, 1412, 1365, 1270, 1161, 1117, 1090, 1003 cm^{-1};

HRMS (ESI): *m/z* calc. for $C_{18}H_{27}NO_3Na^+$ $[M+Na]^+$: 328.1883, found 328.1883.

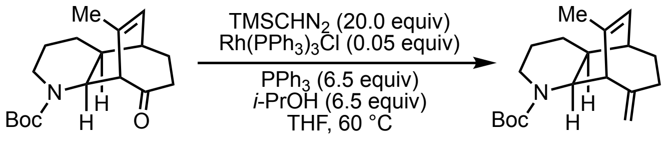

29 **30**

To a mixture of ketone **29** (400 mg, 1.3 mmol, 1.0 equiv), PPh_3 (2.2 g, 8.4 mmol, 6.5 equiv), $Rh(PPh_3)_3Cl$ (60 mg, 0.065 mmol, 0.05 equiv) in THF (43 mL, 0.03 M), *i*-PrOH (0.65 mL, 8.4 mmol, 6.5 equiv) was added. The mixture was heated to 60 °C. $TMSCHN_2$ (43 ml, 26 mmol, 0.6 M in hexanes, 20.0 equiv) was then added dropwise over 10 min. The reaction was stirred for 8 h at 60 °C, then cooled down to room temperature and diluted with Et_2O. The mixture was filtered through a plug of silica and washed with Et_2O. The solvent was removed *in vacuo* and the residue was purified by flash chromatography (Hexane/EtOAc = 60/1) to give alkene **30** (240 mg, 61%) as yellow oil.

Compound **30**:

^{1}H NMR (500 MHz, CDCl$_3$): δ 5.72 (d, J = 7.4 Hz, 1H), 4.78 – 4.65 (m, 2H), 4.46 (d, J = 9.6 Hz, 1H), 3.93 (s, 1H), 2.65 (s, 1H), 2.54 (m, 1H), 2.34 – 2.21 (m, 5H), 1.75 (m, 3H), 1.73 – 1.69 (m, 1H), 1.66 – 1.61 (m, 1H), 1.57 – 1.54 (m, 1H), 1.52 – 1.49 (m, 2H), 1.47 (s, 9H);

^{13}C NMR (126 MHz, CDCl$_3$): δ 155.8, 148.2, 137.8, 125.5, 109.2, 79.4, 56.2, 55.0, 40.0, 38.3, 37.8, 31.6, 29.6, 28.7, 27.5, 21.9, 21.8;

IR (film): 2925, 2858, 1687, 1417, 1390, 1363, 1325, 1252, 1157, 1112, 1080, 1005 cm^{-1};

HRMS (ESI): *m/z* calc. for $C_{19}H_{29}NO_2Na^+$ $[M+Na]^+$: 326.2091, found 326.2101.

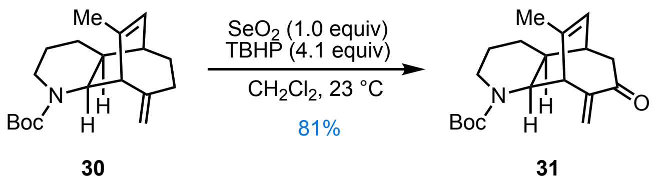

To a stirred solution of alkene **30** (106 mg, 0.35 mmol, 1.0 equiv) in CH_2Cl_2 (7.0 mL) was added TBHP (0.26 mL, 5.5 M in nonane, 1.43 mmol, 4.1 equiv) dropwise followed by adding SeO_2 (38.8 mg, 0.35 mmol, 1.0 equiv). The reaction mixture was stirred for 12 h at room temperature. Upon completion, the crude was subjected to the flash column (Hexane/EtOAc = 4/1) to afford enone **31** (90 mg, 81%.) as yellow oil.

Compound **31**:

^{1}H NMR (500 MHz, $CDCl_3$): δ 6.08 (dt, J = 6.4, 1.8 Hz, 1H), 4.28 (d, J = 10.2 Hz, 1H), 3.96 – 3.87 (m, 1H), 3.12 (s, 1H), 2.84 – 2.76 (m, 1H), 2.47 (ddd, J = 13.3, 10.3, 3.2 Hz, 1H), 2.35 (tdd, J = 9.9, 7.6, 2.6 Hz, 1H), 2.01 (dd, J = 4.1, 2.9 Hz, 2H), 1.87 (m, 1H), 1.80 (d, J = 1.7 Hz, 3H), 1.62 – 1.56 (m, 1H), 1.48 – 1.30 (m, 4H), 1.45 (s, 9H);

^{13}C NMR (126 MHz, $CDCl_3$): δ 200.6, 155.3, 147.8, 139.9, 126.1, 118.8, 80.0, 53.9, 52.9, 45.6, 40.2, 37.8, 36.3, 28.6, 27.4, 23.7, 20.7;

IR (film): 2928, 1685, 1607, 1417, 1364, 1314, 1292, 1270, 1252, 1162, 1145, 1117, 1083 cm^{-1};

HRMS (ESI): m/z calc. for $C_{19}H_{27}NO_3Na^+$ $[M+Na]^+$: 340.1883, found 340.1884.

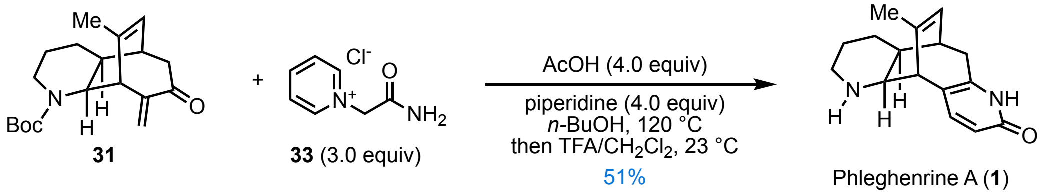

To a solution of enone **31** (40 mg, 0.126 mmol, 1.0 equiv) in n-BuOH (1.3 mL) were added acetic acid (29 µL, 0.5 mol, 4.0 equiv), piperidine (51 µL, 0.5 mmol, 4.0 equiv) and piperidinium salt **33** (65 mg, 0.38 mmol, 3.0 equiv) at room temperature. The mixture was stirred at 120 °C for 48 h before TLC analysis showed full conversion and concentrated *in vacuo*. The crude obtained was dissolved in TFA/CH_2Cl_2 (1/8, 0.4 mL) at room temperature and stirred for 1 h. The reaction was quenched with saturated aq. Na_2CO_3 and extracted with CH_2Cl_2 three times. The combined organic phase was concentrated to give a yellow oil. Column chromatography ($CHCl_3$/MeOH = 10/1 to 7/1) gave phleghenrine A (**1**, 16.4 mg, 51%) as a slightly yellow oil.

Phleghenrine A (**1**):

^{1}H NMR (500 MHz, CD_3OD): *δ* 7.40 (d, *J* = 9.3 Hz, 1H), 6.33 (d, *J* = 9.2 Hz, 1H), 6.01 (d, *J* = 7.1 Hz, 1H), 3.88 (dd, *J* = 9.1, 2.0 Hz, 1H), 3.24 (m, 1H), 3.17 (m, 1H), 3.09 (br s, 1H), 2.83 (dd, *J* = 18.4, 4.0 Hz, 1H), 2.76 (dd, *J* = 18.2, 3.5 Hz, 1H), 2.51 (m, 1H), 2.44 (m, 1H), 1.94 – 1.85 (m, 5H), 1.76 (m, 1H), 1.37 (m, 1H);

^{13}C NMR (126 MHz, CD_3OD): *δ* 165.8, 146.0, 145.5, 142.1, 125.9, 117.8, 116.9, 59.9, 47.3, 41.6, 37.5, 36.5, 36.3, 25.1, 22.4, 18.4;

IR (film): 3450, 2923, 1652, 1610, 1553, 1456, 1178, 1096, 832, 603 cm^{-1};

HRMS (ESI): *m/z* calc. for $C_{16}H_{21}N_2O^+$ $[M+H]^+$: 257.1648, found 257.1651.

Table S1. ^{1}H NMR of **1** and natural sample[4].

position	^{1}H-NMR			
	natural[4]		**Synthetic 1(TFA)**	
1				
2	6.32	d (9.0)	6.33	d(9.2)
3	7.41	d (9.0)	7.40	d(9.2)
4				
5				
6a	2.83	dd (18.6, 3.6)	2.83	dd (18.4, 4.0)
6b	2.76	dd (18.6, 3.6)	2.76	dd (18.2, 3.5)
7	2.50	m	2.51	m
8	6.00	br d (6.6)	6.01	br d (7.1)
9a	3.23	m	3.24	m
9b	3.16	m	3.17	m
10a	1.89	overlap	1.89	overlap
10b	1.75	m	1.76	m
11a	1.89	overlap	1.89	overlap
11b	1.37	m	1.37	m
12	2.43	m	2.44	m
13	3.87	d (8.4)	3.88	dd (9.1, 2.0)
14	3.15	br s	3.09	br s
15				
16	1.87	s	1.88	d (1.6)

Table S2. ^{13}C NMR of **1** and natural sample[4].

position	^{13}C-NMR		
	natural[4]	**Synthetic 1**	$\Delta_{nat-\mathbf{1a}}$
1	165.8	165.8	0.0
2	117.6	117.8	-0.2
3	145.7	145.5	0.2
4	117.1	116.9	0.2
5	146.0	146.0	0.0
6	36.5	36.5	0.0
7	36.2	36.3	0.0
8	125.8	125.9	-0.1
9	41.4	41.6	-0.2
10	18.5	18.4	0.1
11	25.1	25.1	0.0
12	37.5	37.5	0.0
13	59.7	59.9	-0.2
14	47.1	47.3	-0.2
15	142.2	142.1	0.1
16	22.4	22.4	0.0

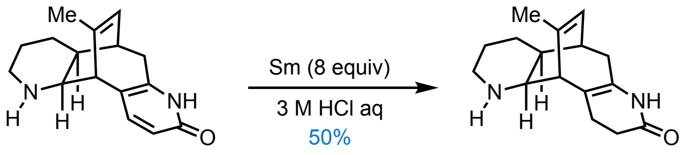

To a solution of phleghenrine A (**1**, 5 mg, 0.019 mmol, 1.0 equiv) in 3 M HCl aq. (0.5 mL) was added Sm metal powder (23 mg, 0.15 mmol, 8 equiv) at 0 °C portionwise over 20 min. The mixture was stirred at 0 °C for 10 min then warmed up to room temperature for 20 min. The crude obtained was quenched with saturated aq. Na_2CO_3 and extracted with CH_2Cl_2 three times. The combined organic phase was concentrated to give a yellow oil. Preparative TLC ($CHCl_3$/MeOH = 10/1) gave phleghenrine C (**3**, 2.5 mg, 50%) as a slightly yellow oil.

Phleghenrine C (**3**):

^{1}H NMR (600 MHz, CD_3OD): δ 5.94 (d, J = 7.2 Hz, 1H), 3.89 (d, J = 9.1 Hz, 1H), 3.24 – 3.14 (m, 2H), 2.56 (s, 1H), 2.50 – 2.43 (m, 4H), 2.43 – 2.34 (m, 2H), 2.27 – 2.17 (m, 2H), 1.89 (d, J = 1.7 Hz, 3H), 1.88 – 1.74 (m, 3H), 1.30 – 1.25 (m, 2H);

^{13}C NMR (150 MHz, CD_3OD): δ 173.2, 142.3, 132.0, 125.1, 111.5, 59.4, 47.5, 41.4, 38.0, 36.8, 36.1, 31.5, 29.1, 25.2, 22.9, 18.6;

IR (film): 3419, 2925, 1664, 1613, 1389, 1176, 1114, 829, 603 cm^{-1};

HRMS (ESI): *m/z* calc. for $C_{16}H_{23}N_2O^+$ $[M+H]^+$: 259.1805, found 259.1807.

Table S3. **^{1}H NMR** of **3** and natural sample[4].

position	^{1}H-NMR			
	natural[4]		Synthetic **3**(**TFA**)	
1				
2	2.43	m	2.45	m
3	2.44	m	2.47	m
4				
5				
6	2.20	m	2.23	m
7	2.32	ddd (6.0, 6.0,6.0)	2.35	m
8	5.90	br d (6.6)	5.94	br d (7.2)
9	3.14	m	3.19	m
10a	1.86	m	1.88	m
10b	1.74	m	1.77	m
11a	1.83	m	1.86	m
11b	1.29	m	1.29	overlap
12	2.36	ddd (12.6, 9.0,6.6)	2.41	m
13	3.82	dd (9.0, 1.8)	3.89	br d (9.1)
14	2.56	br s	2.56	br s
15				
16	1.87	d (1.2)	1.89	d (1.7)

Table S4. ^{13}C NMR of **3** and natural sample[4].

position	^{13}C-NMR		
	natural[4]	**Synthetic 3**	$\Delta_{nat\text{-}\mathbf{1a}}$
1	173.3	173.2	0.1
2	31.5	31.5	0.0
3	29.1	29.1	0.0
4	111.8	111.5	0.3
5	131.8	132.0	-0.2
6	36.1	36.1	0.0
7	36.9	36.8	0.1
8	124.9	125.1	-0.2
9	41.3	41.4	-0.1
10	18.8	18.6	0.2
11	25.4	25.2	0.2
12	38.2	38.0	0.2
13	59.3	59.4	-0.1
14	47.5	47.5	0.0
15	142.5	142.3	0.2
16	23.0	22.9	0.1

Part 2. References

[1] Lai, C.; Shen, Y.; Wang, M.; Rao, N. S. K.; Liao, C. *J. Org. Chem.* **2002**, 67, 6493.

[2] Magnus, P.; Seipp, C. *Org. Lett.* **2013**, *15*, 4870.

[3] Yu, J.; Truc, V.; Riebel, P.; Hierl, E.; Mudryk, B. *Tetrahedron Lett.* **2005**, *46*, 4011.

[4] Dong, L.; Wu, X.; Shi, X.; Zhang, Z.; Yang, J.; Zhao, Q. *Org. Lett.* **2016**, 18, 4498.

Part 3. ^{1}H and ^{13}C NMR spectra

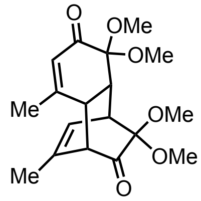

^{1}H NMR (500 MHz, $CDCl_3$)

21

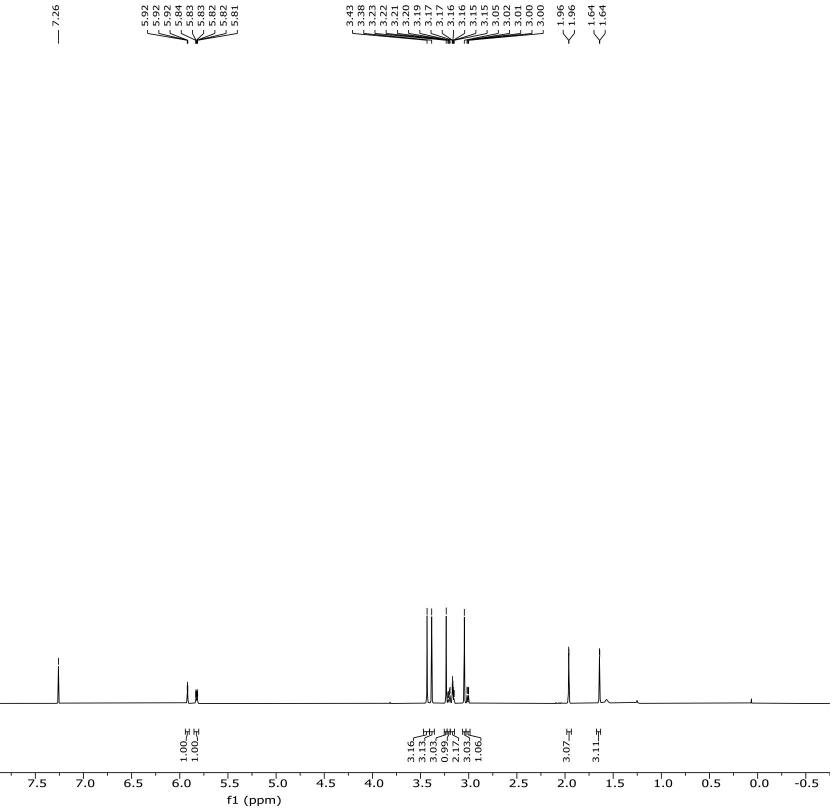

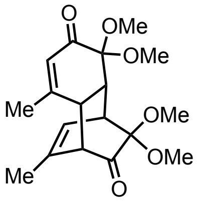

^{13}C NMR (126 MHz, $CDCl_3$)

21

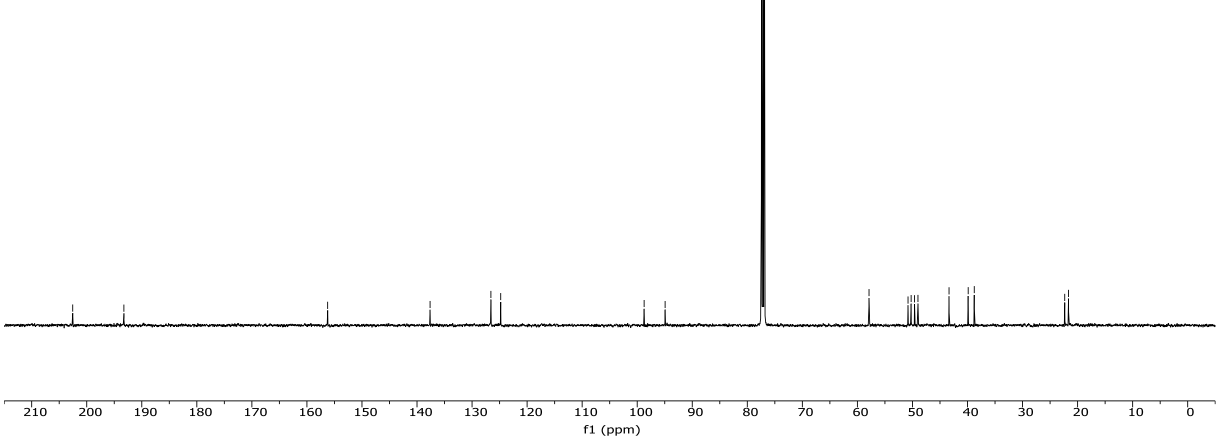

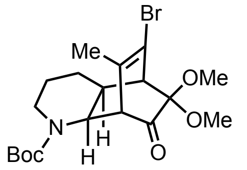

^{1}H NMR (500 MHz, $CDCl_3$)

26

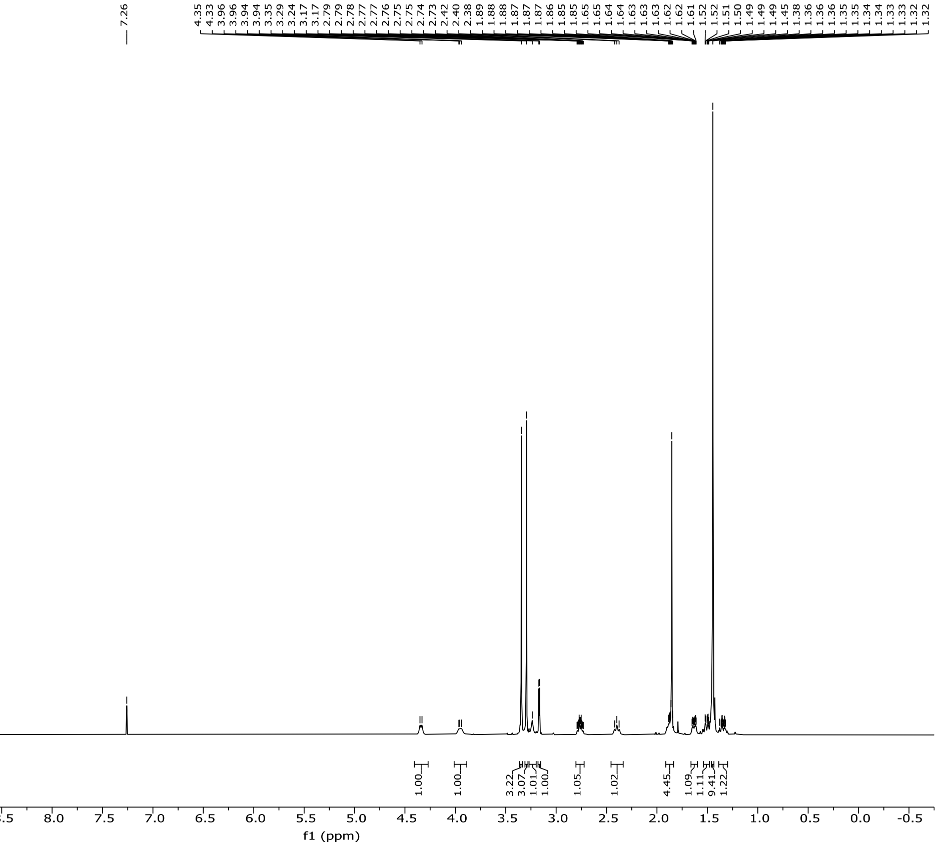

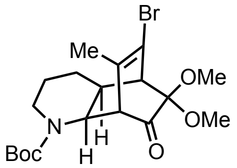

^{13}C NMR (126 MHz, $CDCl_3$)

26

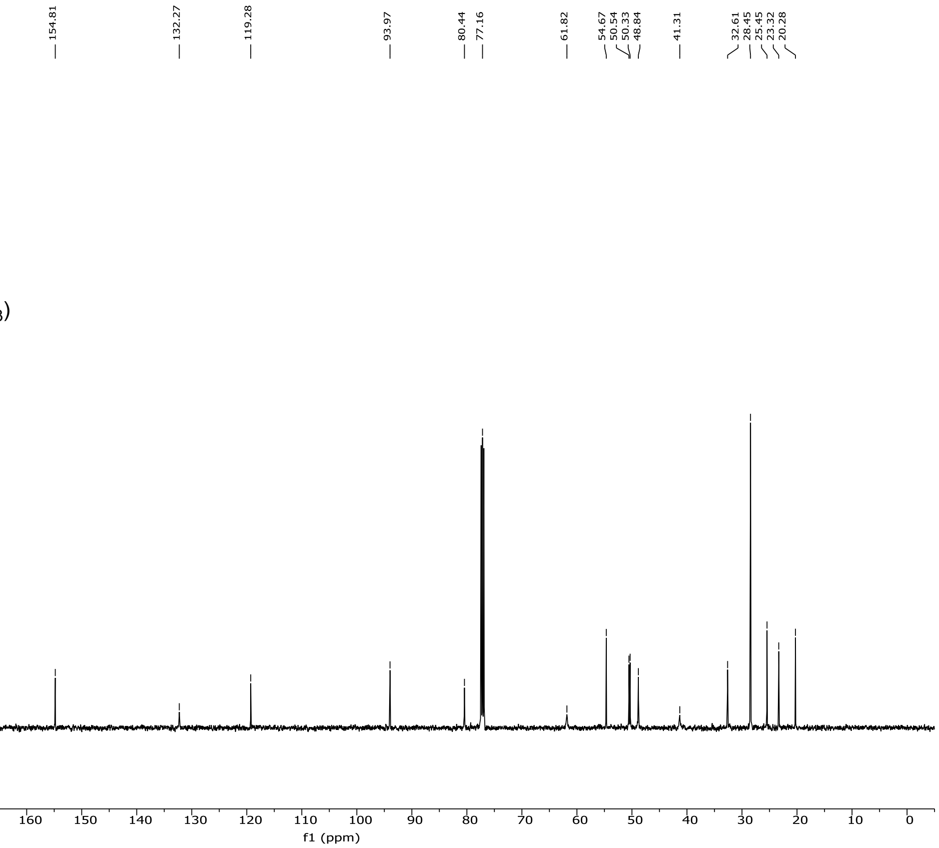

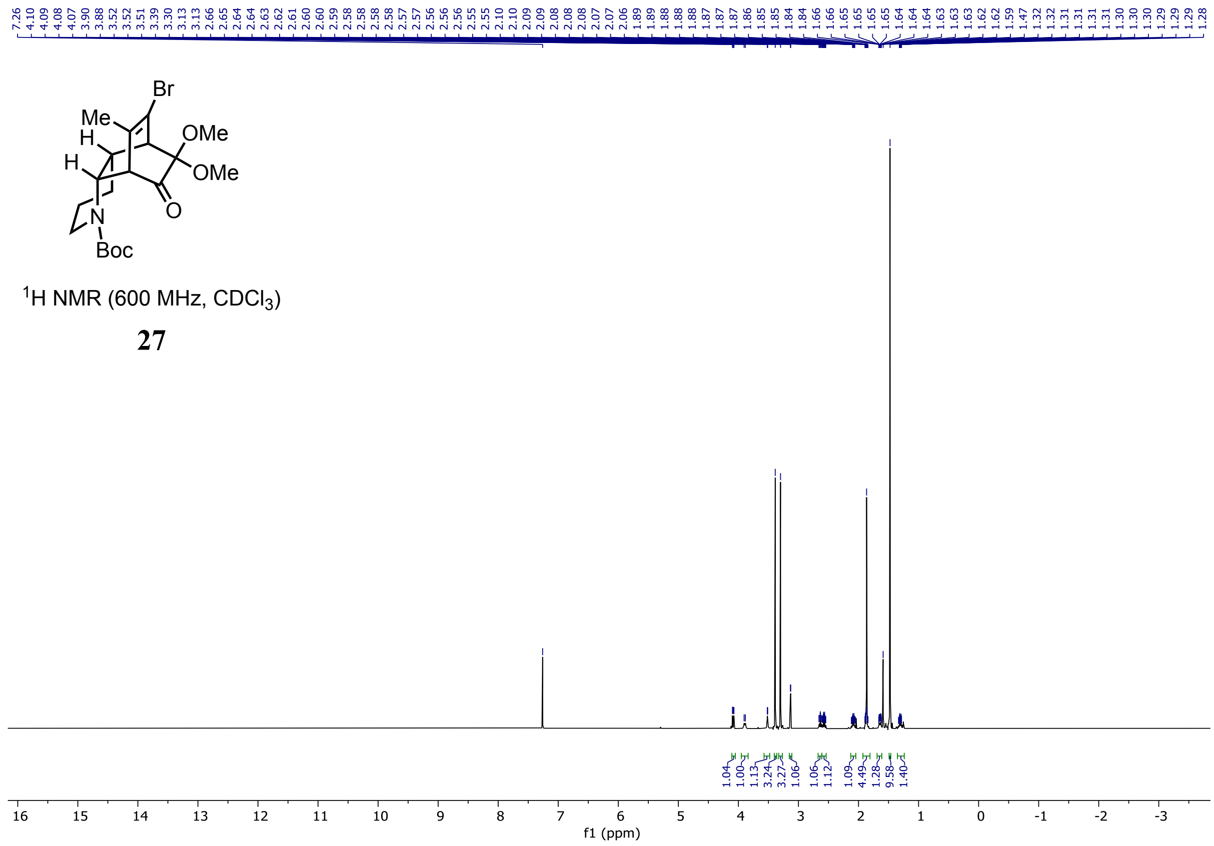

^{1}H NMR (600 MHz, $CDCl_3$)

27

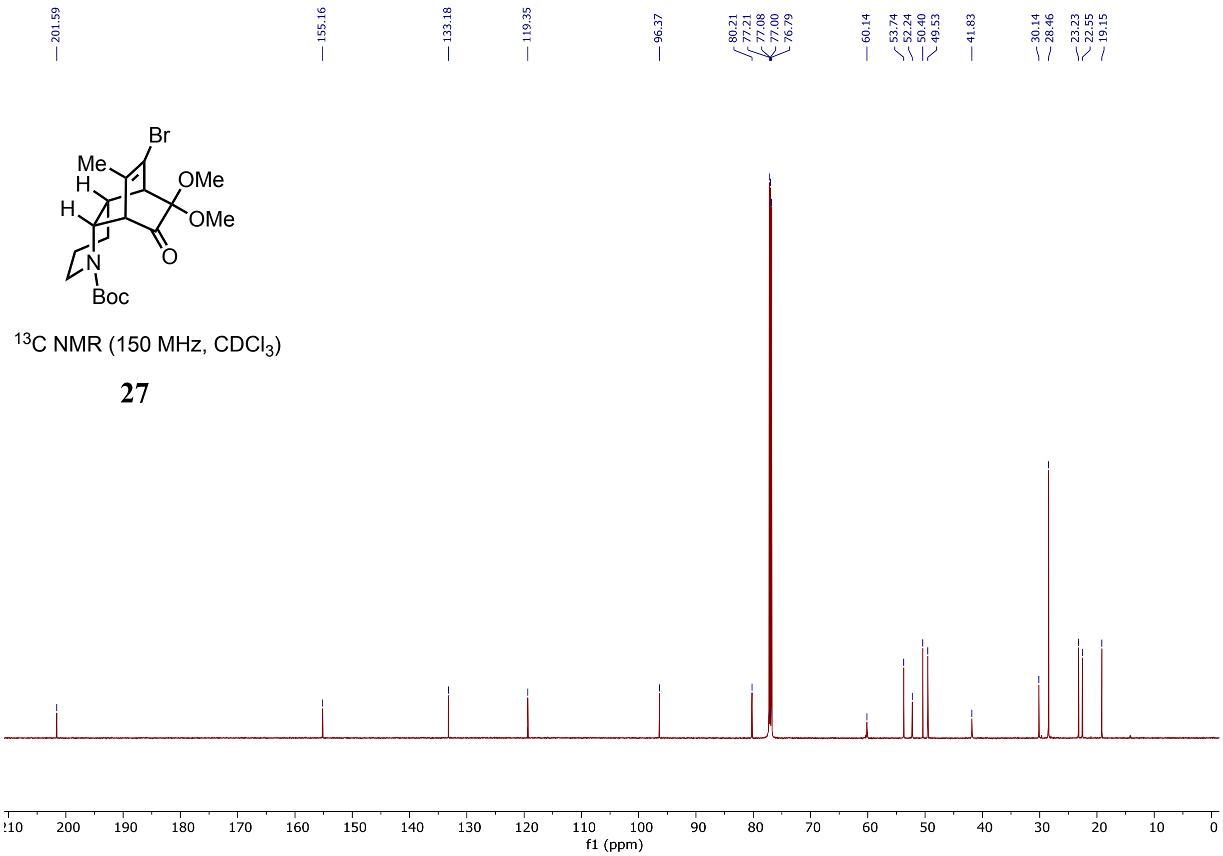

^{13}C NMR (150 MHz, $CDCl_3$)

27

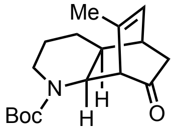

^1H NMR (500 MHz, $CDCl_3$)

28

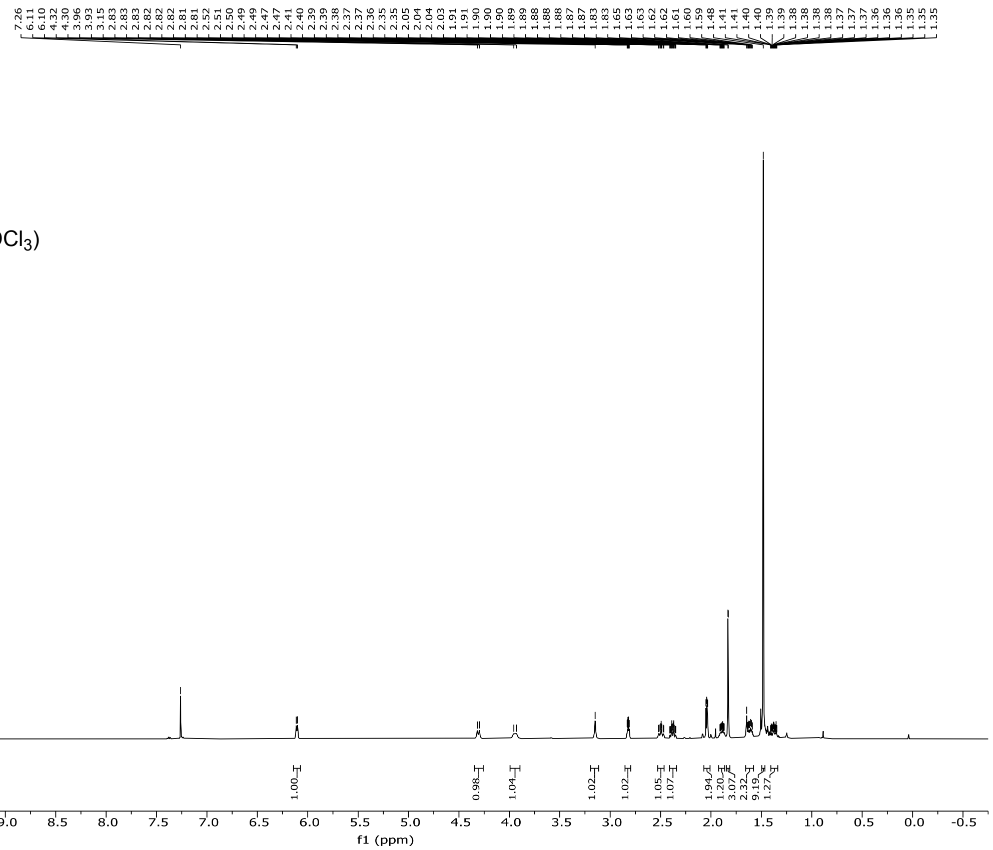

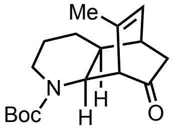

^{13}C NMR (126 MHz, $CDCl_3$)

28

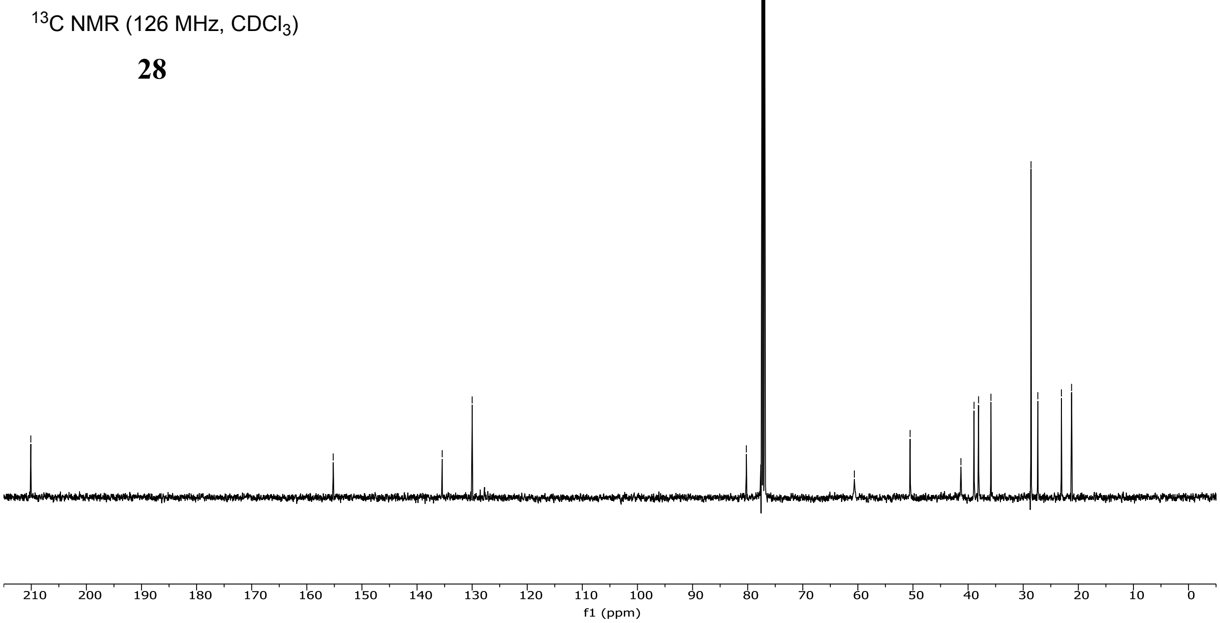

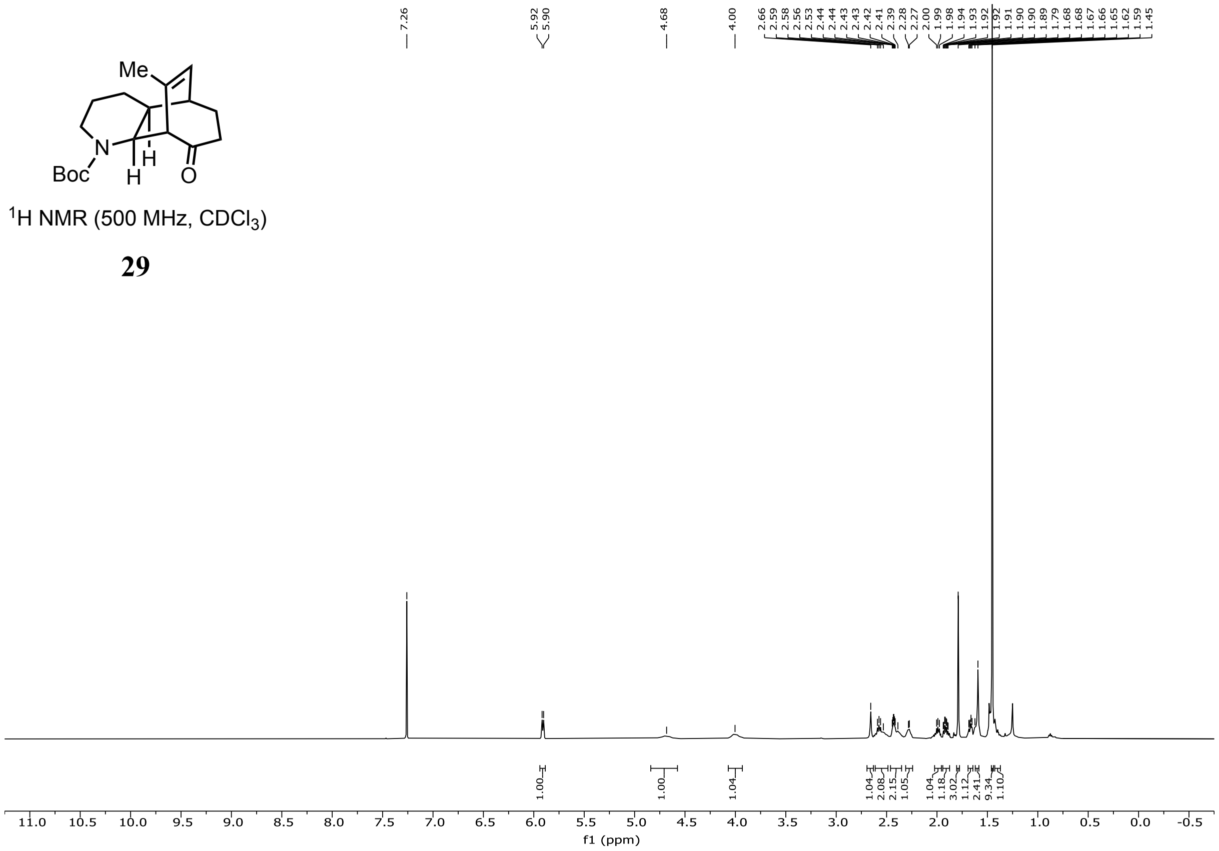

^{1}H NMR (500 MHz, $CDCl_3$)

29

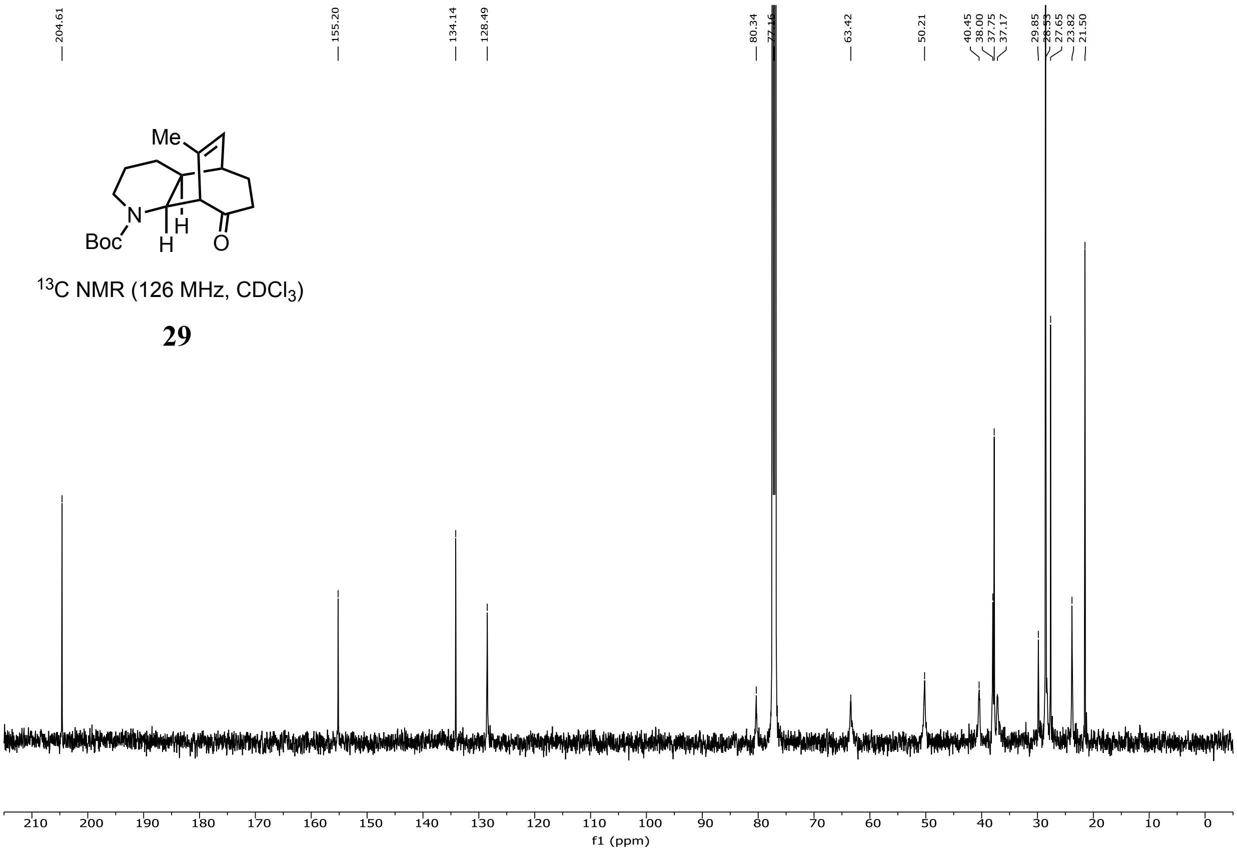

^{13}C NMR (126 MHz, $CDCl_3$)

29

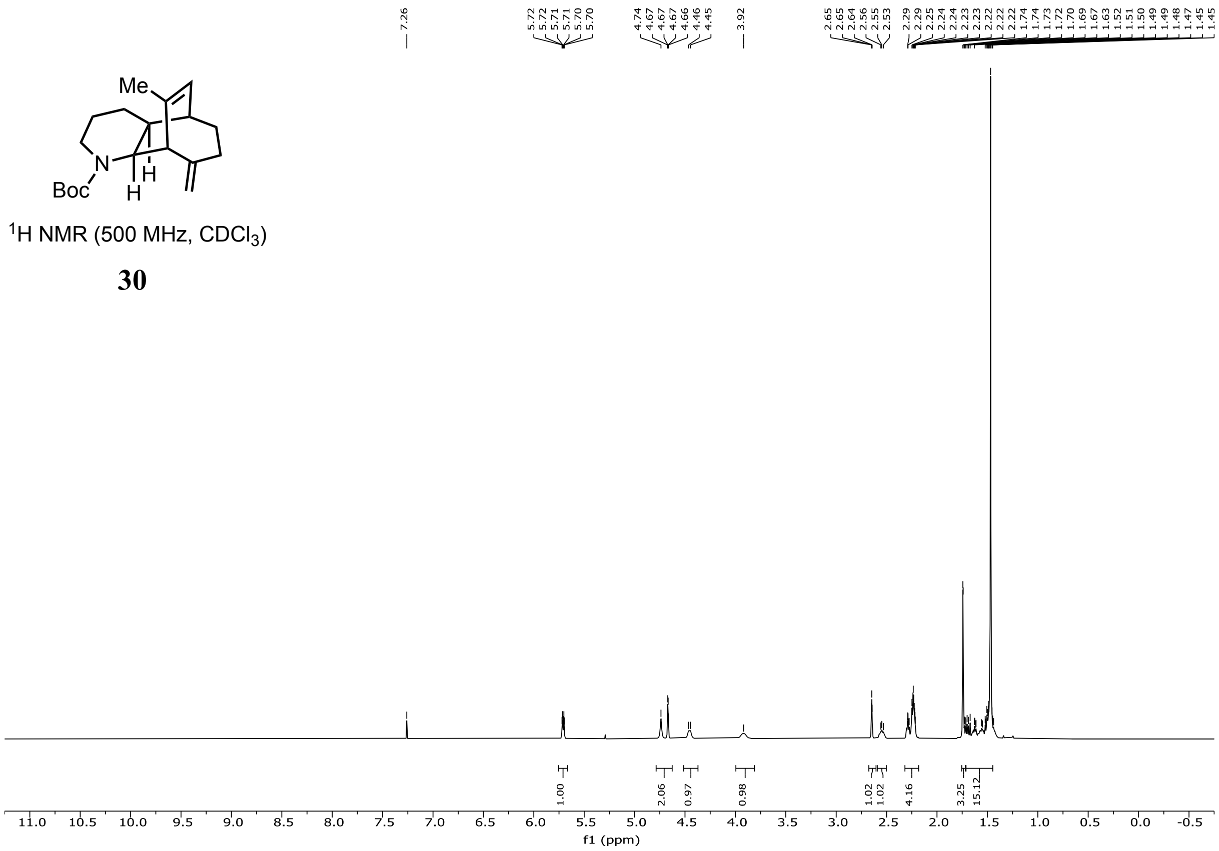

^{1}H NMR (500 MHz, $CDCl_3$)

30

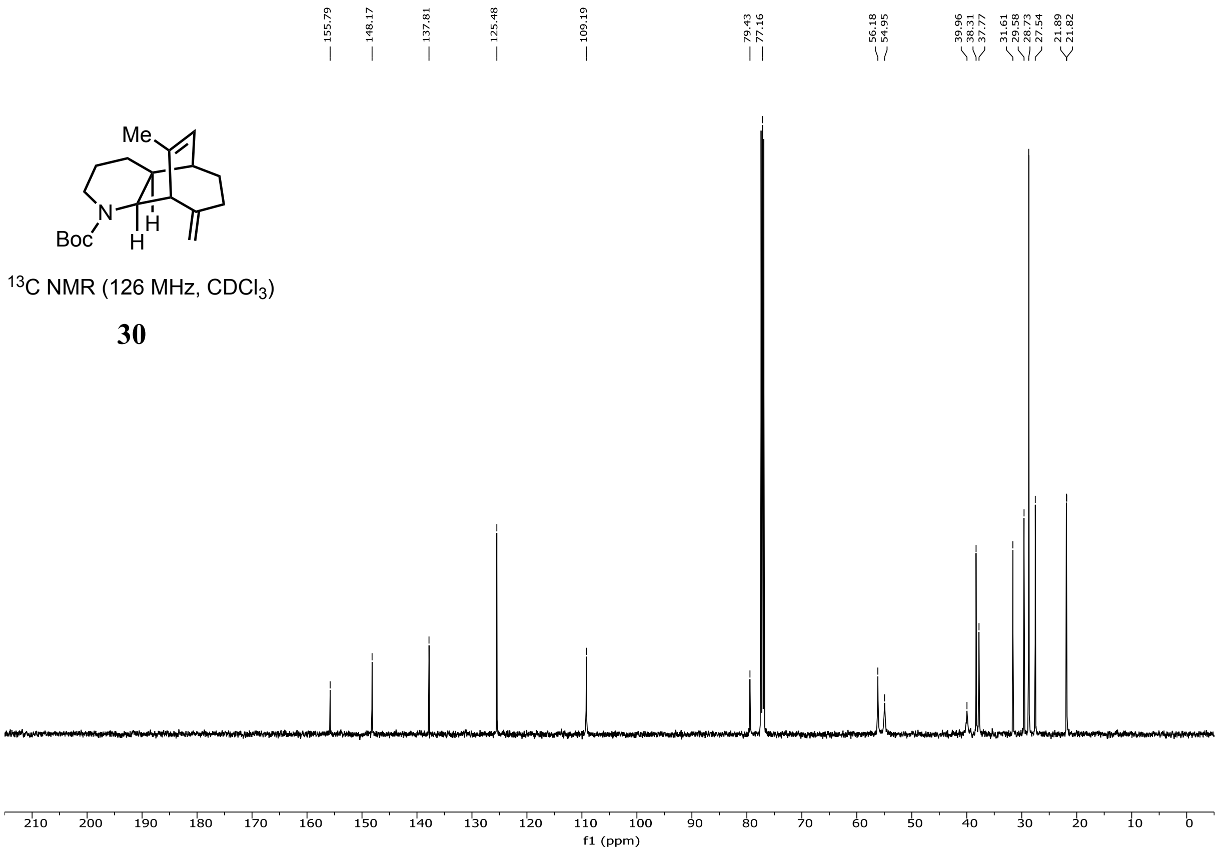

^{13}C NMR (126 MHz, $CDCl_3$)

30

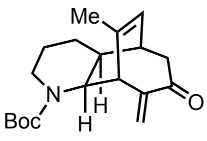

^1H NMR (500 MHz, $CDCl_3$)

31

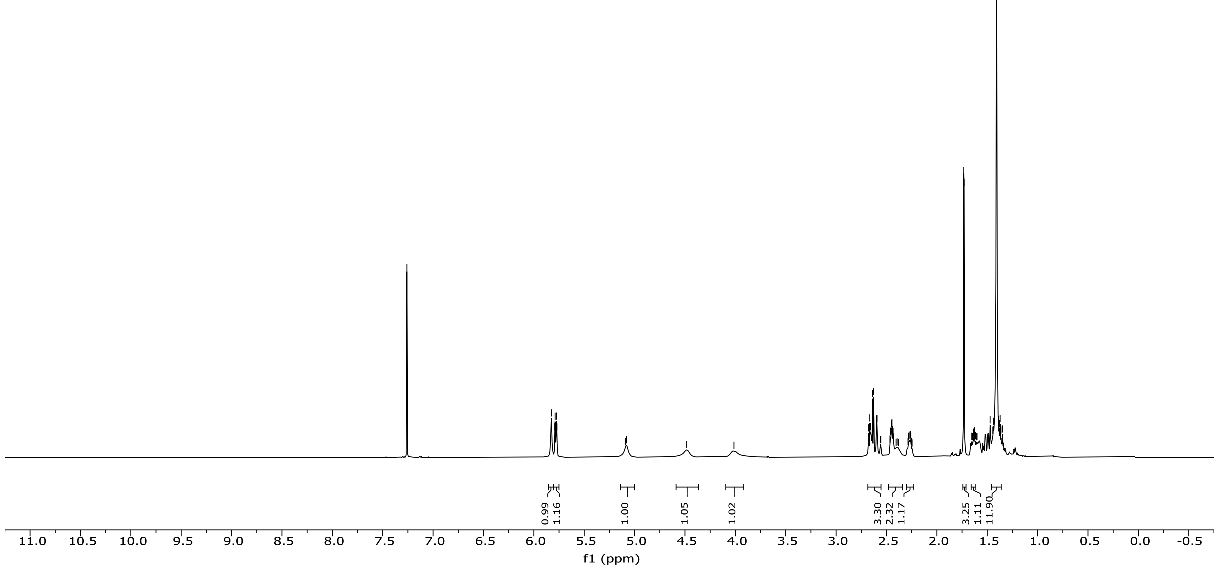

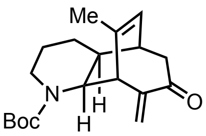

^{13}C NMR (126 MHz, $CDCl_3$)

31

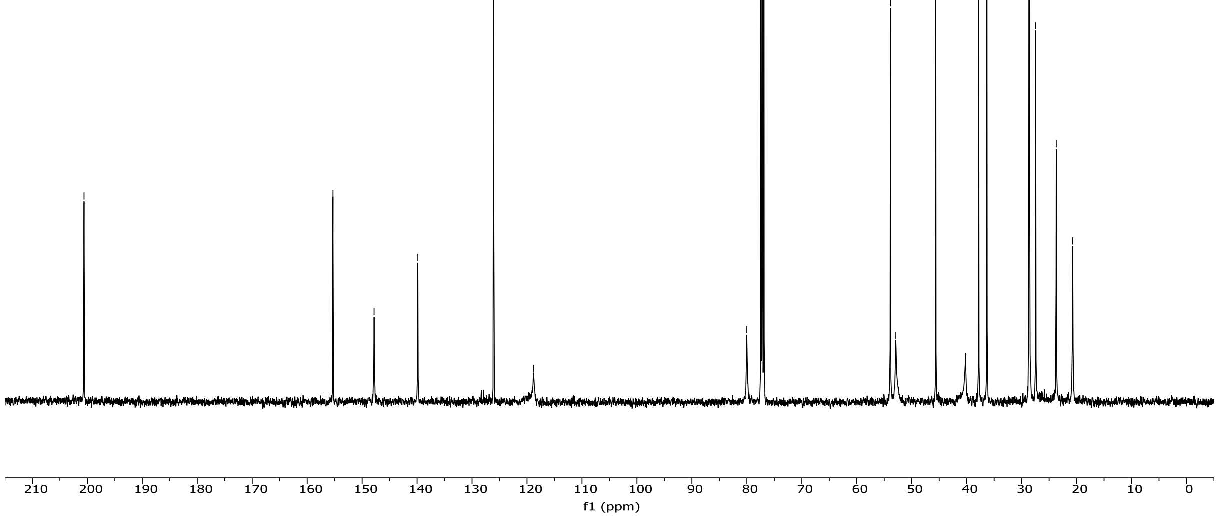

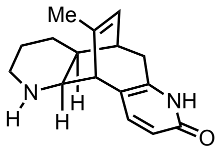

Phleghenrine A
^{1}H NMR (500 MHz, MeOD)

1

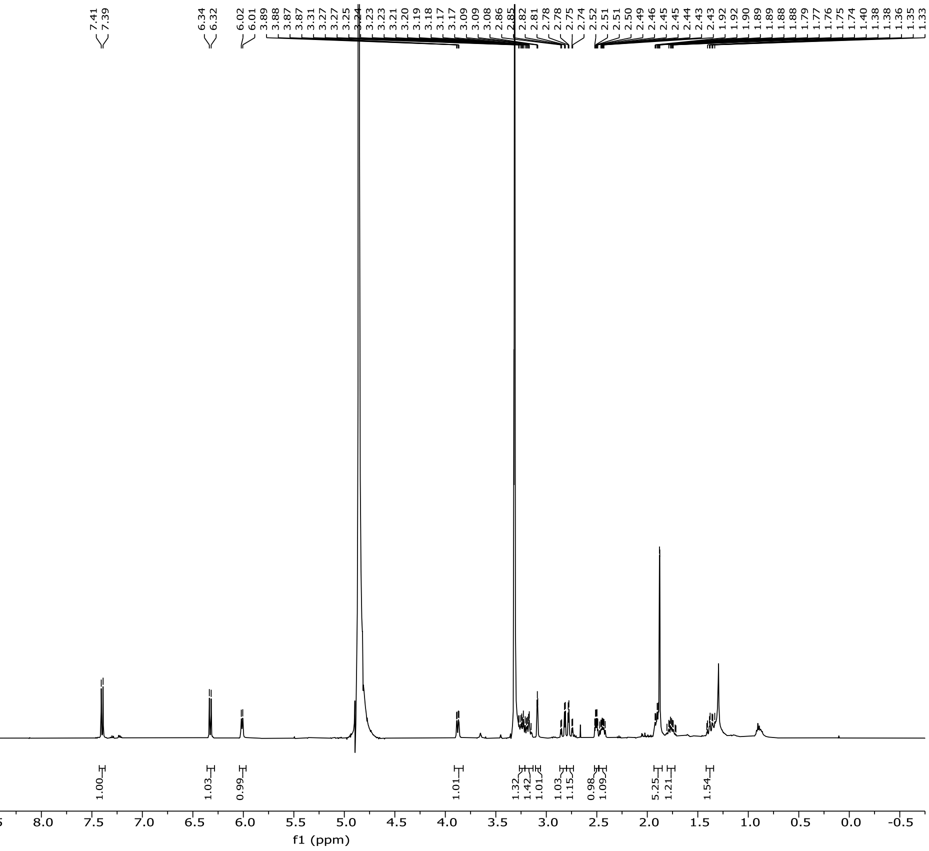

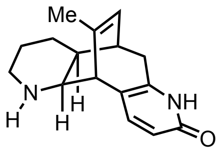

Phleghenrine A
^{13}C NMR (126 MHz, MeOD)

1

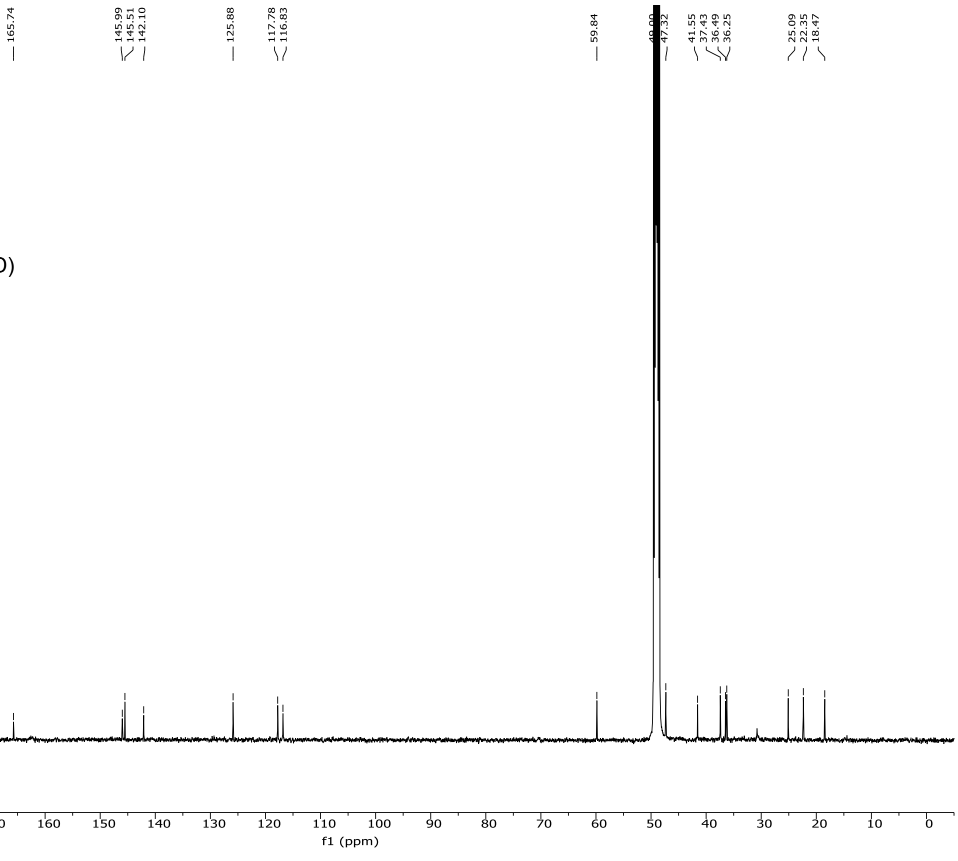

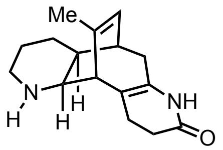

Phleghenrine C
^{1}H NMR (600 MHz, MeOD)

3

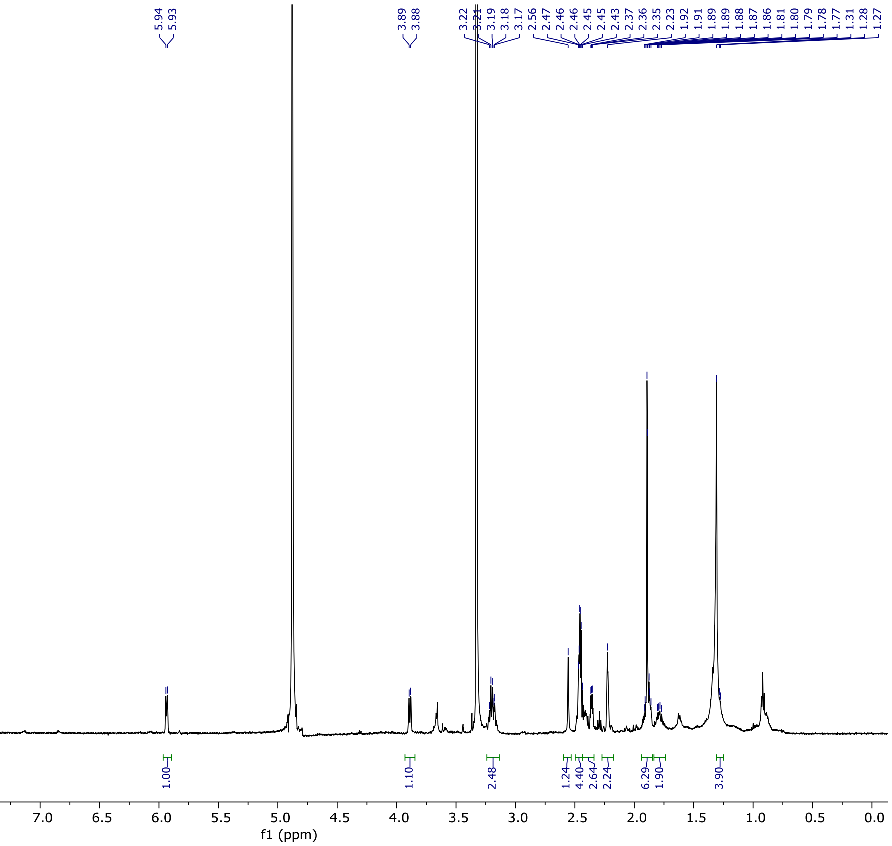

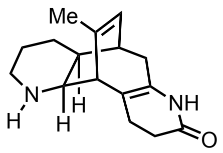

Phleghenrine C
^{13}C NMR (150 MHz, MeOD)

3

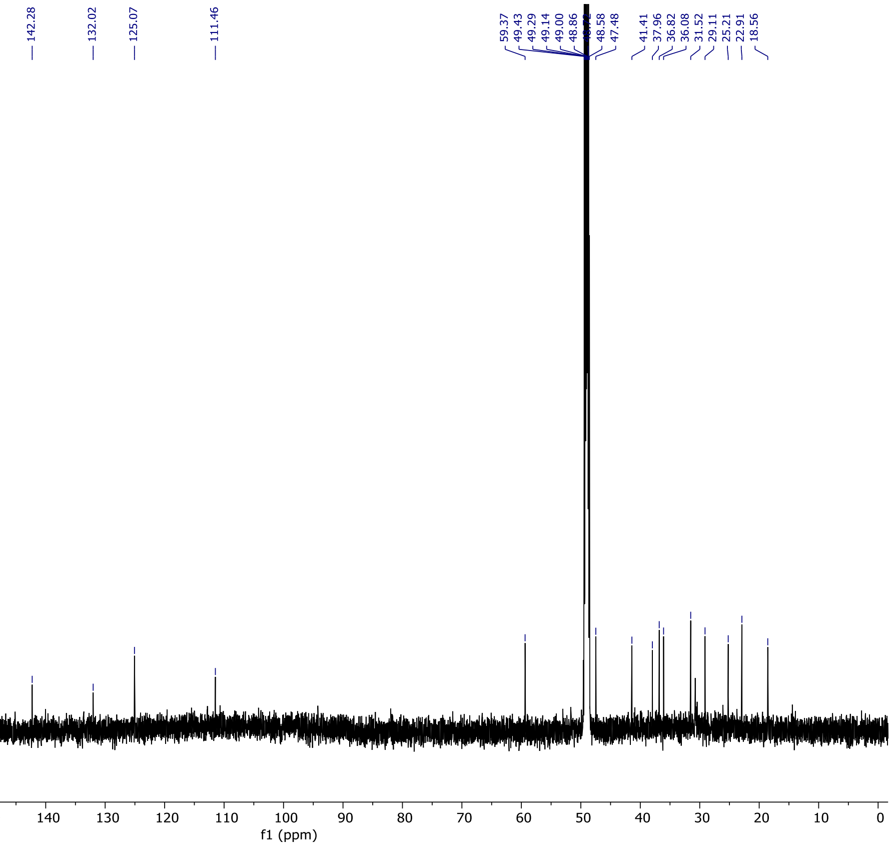

Supplement: Supplementary file 1 — ol3c01784_si_001.pdf [file ol3c01784_si_001.pdf]
